# The Expansion of CD25^high^IL-10^high^FoxP3^high^ B Regulatory Cells Is in Association with SLE Disease Activity

**DOI:** 10.1155/2015/254245

**Published:** 2015-10-04

**Authors:** Zahava Vadasz, Regina Peri, Nasren Eiza, Gleb Slobodin, Alexandra Balbir-Gurman, Elias Toubi

**Affiliations:** ^1^Division of Allergy & Clinical Immunology, Bnai Zion Medical Center, Faculty of Medicine, Technion, 4940 Haifa, Israel; ^2^Rheumatology Unit, Bnai Zion Medical Center, Faculty of Medicine, Technion, 4940 Haifa, Israel; ^3^B. Shine Rheumatology Unit, Rambam Health Care Campus, Faculty of Medicine, Technion, 4940 Haifa, Israel

## Abstract

B regulatory cells (Bregs) belong to a subgroup of activated B cells tasked with maintaining self-tolerance and preventing autoimmunity. While sharing similar regulatory mechanisms such as IL-10 dependency, they also defer in exhibiting their suppressive effects by expressing Fas-Ligand, TGF-beta, and PDL-1. In this study we show, for the first time, the expansion of CD25^high^FoxP3^high^ Bregs in systemic lupus erythematosus (SLE) patients compared to healthy individuals (18.5 ± 3.052% versus 11.0 ± 1.654%, *p* < 0.001, resp.). This expansion was also shown to correlate with SLE disease activity (*r* = 0.75). In addition, CD25^high^FoxP3^high^ Bregs were also IL-10^high^ expressing and further expanded when stimulated with semaphorin 3A. In sum we show that CD25^high^FoxP3^high^ are an additional subtype of Bregs, involved in regulating SLE disease activity. Being IL-10 expressing, we may assume that they are one of the sources of increased serum IL-10 in SLE patients. Further studies are required in order to assess the relation between high serum IL-10 and CD25^high^FoxP3^high^ Breg cells.

## 1. Introduction

Among the many immune mediated responses involved in systemic lupus erythematosus (SLE) is the imbalance between T-helper cells (Th) subsets, namely, Th1/Th2/Th17, and both T and B regulatory (reg) cells [1]. Th1 proinflammatory cytokine levels such as IL-12, IL-6, and IFNs are usually increased in association with SLE disease activity index (SLEDAI). Th17 related cytokines such as IL-17 and IL-21 are also reported to be enhanced and contribute to inflammatory processes in SLE and other rheumatic diseases such as rheumatoid arthritis (RA) and psoriasis. Th2 related cytokines, that is, IL-4 and IL-10, are known for their ability in driving humoral immune responses, B cell overactivation, and the production of many specific autoantibodies [2–5]. Many studies during the last decade have reported on the failure of Treg cells to maintain self-tolerance, allowing the development of many autoimmune diseases. The failure in suppressing effector Th cell proliferation is mainly considered to be IL-10 dependent (lower expression and/or production of IL-10) due to the altered expression of FoxP3 and/or inhibitory molecules such as CTLA-4 in Treg cells [6]. Breg cells are involved in regulating/suppressing immune mediated inflammation but act earlier than Treg cells. They use similar suppressive modalities, that is, IL-10, TGF-beta, and the expression of proapoptotic membrane molecules which vary across different Breg subtypes [7]. Among these different subtypes, CD19^+^CD24^high^CD38^high^ and CD19^+^CD25^high^CD86^high^CD1d^high^ were both described as being involved in suppressing autoimmune processes, both in an IL-10 dependent way and with an altered function in SLE [8, 9]. Breg cells have also been characterized as CD5^high^, FoxP3^high^, and Fas-Ligand expressing cells. CD19^+^CD5^high^FoxP3^high^ Breg cells were reported to be involved in non-IgE-mediated food allergies, namely, in maintaining tolerance to milk allergies [10]. In addition to this subtype, Breg cells were defined as being CD19^+^CD5^high^Fas-L^high^, also called “killer B cells.” Numerous researchers have reported that these cells participate in the escape of viral infections from the efficient cytotoxic T cell response [11]. The similarities and differences between all the above-mentioned Breg cells are not sufficiently understood. Are they similar in their regulatory effects? Do they express/produce similar amounts of IL-10 and TGF-beta? How do they react to various stimuli? (see [12]). In previous studies, we and others showed that Breg cell function was enhanced when stimulated by CpG and CD40L, increasing by this autologous Treg cell properties following their coculture [9, 13]. When cocultured with semaphorin 3A (sema3A), IL-10 and TGF-beta expression was enhanced in CD19^+^CD25^high^ Breg cells, suggesting that sema3A is a frontier factor in improving Breg cell function (unpublished data). Later, we reported on the ability of sema3A in enhancing Breg cell properties by increasing CD72 (a regulatory molecule) expression on B cells [14]. Expecting to find lower serum levels of IL-10 in some autoimmune diseases, namely, in SLE, the opposite was found. Paradoxically, serum IL-10 is reported to be increased in association with increased SLEDAI and with high titers of anti-dsDNA antibodies. The source of increased serum IL-10 in SLE is yet undefined, suggested to be overproduced by Th2 and/or by one of the Breg subtypes. In addition, the association of Atg5 rs573775 single nucleotide polymorphism (SNP) with SLE susceptibility and IL-10 serum levels was analyzed. Here, carriage of the rs573775 T allele was associated with IL-10 upregulation and clinical features of SLE, concluding that such mutated allele influenced both SLE susceptibility and IL-10 production [15]. In this study, we aim to evaluate the status of CD19^+^CD25^high^FoxP3^high^ Breg cells, namely, whether they are IL-10 expressing. We will also assess the status of these cells in SLE patients when compared to healthy individuals. We speculate on their possible contribution to increased serum IL-10 in SLE patients. Finally, we will evaluate the response of this subtype of Breg cells to sema3A, to see if this coculture increases IL-10 expression as it does in other Breg cells.

## 2. Patients and Methods

### 2.1. Patients Population

This study examined 21 SLE patients (20 females and 1 male; age range 16–59 years; mean 30.5 ± 9.2). All patients are routinely followed up by well-trained rheumatologists and all fulfill the ACR criteria for the classification of SLE [16]. Clinical and serological data (skin involvement; arthritis; renal involvement; full cell blood count; serum complement levels; anti-dsDNA and other extractable nuclear autoantibodies) were all available, enabling the determination of SLEDAI. The serological work-up was performed at the Bnai Zion Medical Center by a single experienced technician to insure uniformity of all analyses, utilizing identical kits. Patients in whom SLEDAI was between 4 and 6 points were treated with hydroxychloroquine and in some patients prednisolone (2.5 mg/daily) was added. When SLEDAI was above 7 points, azathioprine was added, but only after analyzing specific serology and purifying B cells. When SLEDAI was above 12 points the addition of cyclophosphamide or MMF was considered again, only after performing SLE serology and purifying B cells. Twenty healthy controls, sex and age matched, were assessed and analyzed for all above-mentioned parameters. This study was approved by both the local Helsinki Committee of the Bnai Zion Medical Center and the Rambam Health Care Campus, Haifa, Israel.

### 2.2. B Cell Purification

B cells were purified from peripheral blood of healthy controls and SLE patients. To do so, peripheral blood mononuclear cells (PBMCs) were isolated on Lymphoprep (Axis-Shield, Oslo, Norway), and B lymphocytes were then twice purified by positive selection using CD22 microbeads (20 *μ*L/10^7^ cells; Miltenyi Biotec, Bergisch Gladbach, Germany) according to the manufacturer's instructions, achieving by this >99% purity.

### 2.3. FoxP3 and IL-10 Expression in CD19^+^CD25^high^ B Cells

The expression of FoxP3 and IL-10 in CD19^+^CD25^high^ cells (considered as Breg cells) from healthy controls and SLE patients was initially assessed by staining purified B cells after 48 hours of activation with ODN-CpG and CD40L. The staining was performed by using monoclonal antibodies, human anti-CD19-BUV737 (BD Horizon, Becton Dickinson, NJ, USA) and human anti-CD25 BUV395 (BD Horizon, Becton Dickinson, NJ, USA) as outer membrane antibodies, and FoxP3 PE∖CF594 and IL-10 APC (BD Horizon, Becton Dickinson, NJ, USA) as intracellular staining, using a “Fix and Perm” kit (Invitrogen, NY, USA) according to the manufacturer's instructions. The staining was evaluated using flow cytometry software (FC500 and CXP software, Beckman Coulter, Brea, CA, USA, and Becton Dickinson, NJ, USA). CD3 positive cells in the purified cell culture were determined by using monoclonal CD3 PerCP-Cy5.5 antibody (BD Pharmingen, Becton Dickinson, NJ, USA) and analyzed by Becton Dickinson FACS-Fortessa. The results are shown as % of CD19^+^CD25^high^ Breg cells expressing FoxP3 or IL-10, taking into consideration that the absolute number of Breg cells in all groups was found to be comparable. Standard deviation (STDEV) was used to quantify the amount of variation of a set of data values (e.g., percentage of Breg cells expressing FoxP3 among the patients in each indicated group of disease or normal control).

### 2.4. Semaphorin 3A Enhances FoxP3 Expression

Aiming to evaluate the effect of sema3A on FoxP3 expression, condition-media from HEK293− cells, which were infected by NSPI-CMV-FLAG lentivirus with or without human sema3A cDNA, a kind gift from Professor Gera Neufeld and Dr. Ofra Kessler, Ruth and Bruce Rappaport Faculty of Medicine, Technion, Israel, as previously described [17], were added to the above-mentioned purified B cells activated by ODN-CpG and CD40L and incubated for 48 hours. After incubation, CD19^+^CD25^high^ cells were analyzed for the possible change in FoxP3 expression using the above-mentioned specific monoclonal antibodies and evaluated using an FC500 flow cytometer and Becton Dickinson FACS-Fortessa. The results are shown as % of Breg cells expressing FoxP3, taking into consideration that the absolute number of Breg cells in all groups was found to be comparable.

### 2.5. Clinical Correlation and Statistical Analysis

Comparison of FoxP3 expression in B cells from SLE patients and healthy controls was done using the unpaired Student *t*-test. The correlation coefficient (*r*) of clinical correlation between SLEDAI score and % of Breg cells expressing FoxP3 was determined using the Pearson correlation test. A two-tailed *p* value of 0.05 or less was considered to be statistically significant.

## 3. Results

### 3.1. CD19^+^CD25^high^ Activated B Cells Are FoxP3^high^

**Figure 1:**
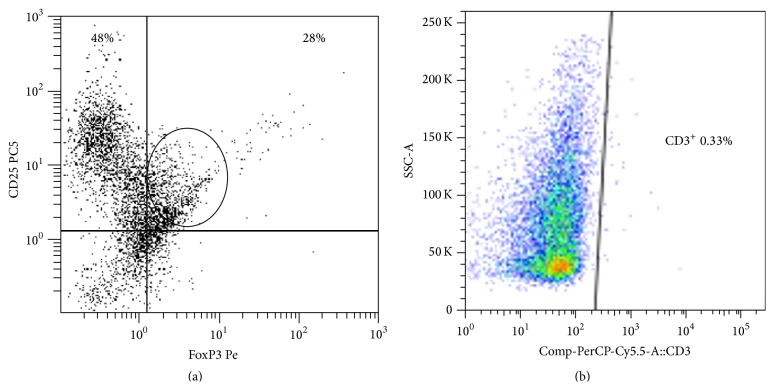
(a) A representative FACS analysis of purified B cells (expressing CD25^high^ FoxP3) following CpG-ODN and CD40L activation. Of note is that CD25^dim-low^ B cells (upper left quadrant) do not express FoxP3. However, CD25^high^ B cells coexpress significant amount of FoxP3 (upper right quadrant). (b) A representative FACS analysis of purified activated B cells, showing that CD3^+^ T cell contamination (gated CD3^+^ T cells) is less than 0.5%.

First, we examined whether CD19^+^CD25^high^ B regulatory cells are also FoxP3 expressing cells. Purified resting B cells (immediately following purification) were FoxP3^dim^ (weakly detectable) (data not shown). However, following their stimulation with CpG-ODN and CD40L for 48 h, CD19^+^CD25^high^ B cells turned to become FoxP3^high^ (Figure 1(a)). As also seen, there are less than 0.5% gated CD3 T cells and therefore B cell contamination with CD3 is unlikely and FoxP3 expression in CD25^high^ B cells is very prominent (Figure 1(b)).

### 3.2. Activated CD19^+^CD25^high^ FoxP3^high^ Are Also IL-10^high^

**Figure 2:**
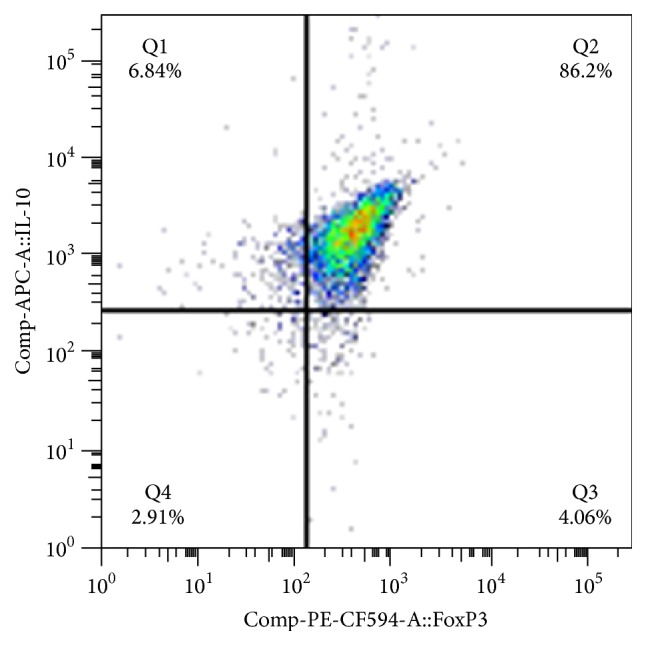
A demonstrative FACS analysis of purified B cells, showing that activated CD19^+^CD25^high^FoxP3^high^ are also IL-10^high^. Of note is that CD25^dim-low^∖FoxP3^dim-low^ B cells express very little IL-10.

Gating on activated CD25^high^FoxP3^high^ one can see that most of these cells (>85% of these cells) are IL-10^high^ (Figure 2) in contrast to B cells that are FoxP3^dim^ being also IL-10^dim^.

### 3.3. CD19^+^CD25^high^FoxP3^high^ in SLE

**Figure 3:**
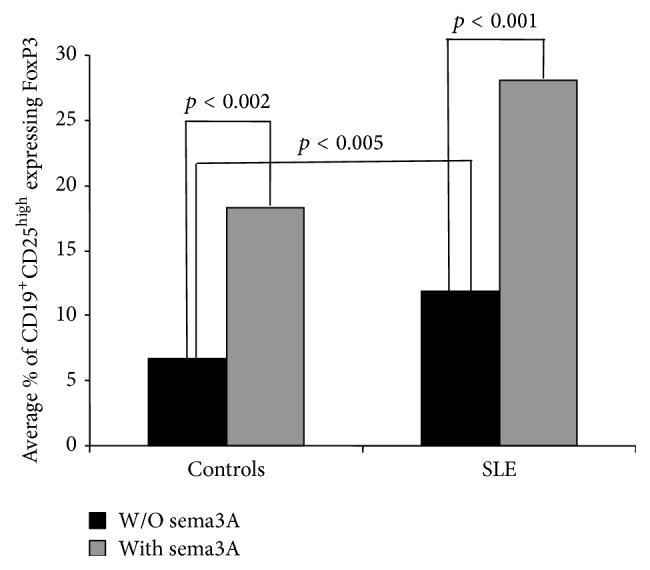
The percentage of CD19^+^CD25^high^FoxP3^high^ Breg cells in normal controls (*n* = 20) and in patients suffering from SLE (*n* = 21). One can see that this subtype of B cells is significantly increased in SLE patients. In addition, the addition of sema3A to these cells increased significantly the percentage of these cells.

The percentage of Breg cells (CD19^+^CD25^high^ cells) in peripheral blood (highly expressing FoxP3) was significantly higher in SLE patients when compared to that of healthy individuals (18.5% ± 3.052 versus 11.0 ± 1.654%, resp., *p* < 0.005) (Figure 3).

### 3.4. Semaphorin 3A Increases FoxP3 Expression in Breg Cells

We then sought to determine if sema3A increases the expression of FoxP3 in these Breg cells. As is demonstrated in Figure 3, sema3A increases the percentage of Breg cells (CD19^+^CD25^high^ cells) in peripheral blood expressing FoxP3, in normal controls and to a higher extent in SLE patients (in normal controls up to 13.6 ± 1.806% from baseline, *p* < 0.002, and in SLE patients up to 28.5 ± 3.506%, *p* < 0.0001) (Figure 3).

### 3.5. FoxP3 Expression in B Cells Is Correlated with SLEDAI

**Figure 4:**
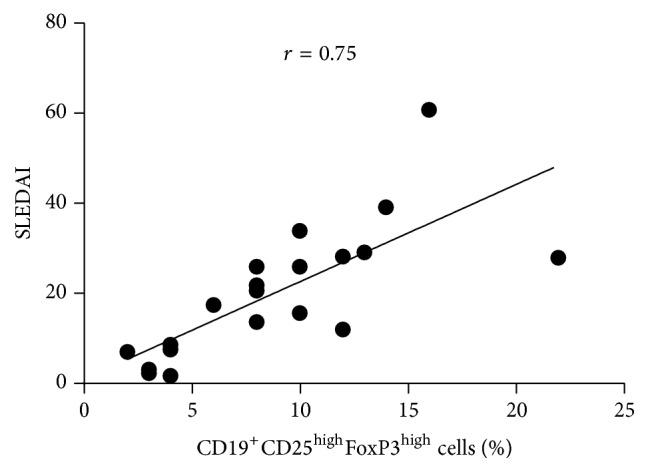
Clinical correlation between the percentage of CD19^+^CD25^high^FoxP3^high^ cells of SLE patients and the SLEDAI score of these patients. The correlation was done using the Pearson correlation test.

Figure 4 demonstrates the correlation between the percentage of CD19^+^CD25^high^FoxP3^high^ cells of SLE patients and the SLEDAI score of these patients. As can be seen, there is a positive correlation with an “*r*” Pearson coefficient of 0.75. This result is in line with the known correlation between IL-10 level in SLE patients and their SLEDAI.

## 4. Discussion

In most autoimmune diseases, immune mediated inflammatory damage is always the result of a net balance between the overactivity of self-reactive cells (T and B effector cells) and immune regulatory mechanisms (T and B regulatory cells). Most B regulatory cells are defined as being IL-10 expressing/producing cells; however, they have different subtypes, are heterogeneous, and have different mechanisms in diseases in which they are involved. Their homology to Treg subtypes, namely, Br1 cells (expressing IL-10), Br3 cells (mainly expressing TGF-beta), and B-FoxP3 positive cells, was recently mentioned. In this case, Breg cells were shown to initiate immune regulatory responses by facilitating the recruitment of Tregs and then disappearing once Tregs become dominant in the immune response [18]. As mentioned above, when CD19^+^CD24^high^CD38^high^ B cells were evaluated in SLE, they had both a reduced ability to produce IL-10 and a reduced ability to suppress T cell cytokine production, although it is unclear if this latter defect is a cause or a consequence of SLE. In contrast to this finding, human IL-10 producing CD24^high^CD27^high^ Breg cells (found to suppress monocytes in an IL-10 dependent manner) were increased in patients with rheumatoid arthritis, SLE, and multiple sclerosis when compared to healthy individuals, suggesting this increase to be compensatory, aiming (with little success) to maintain self-tolerance [19]. The role of CD5^high^FasL^high^ “killer B cells” was assessed in lupus susceptible MRL/lpr mice. Being cytotoxic to T cells they were found to be increased, probably in attempt to suppress autoreactive T cells in these mice [20]. Focusing on CD19^+^CD25^high^FoxP3^high^ Breg cells we first assessed their status in healthy individuals. Here, we show for the first time that both IL-10 and FoxP3 expressions were noticed mainly in activated CD25^high^ B cells (activated with CpG and ODN) and that this expression was enhanced when these B cells were stimulated with add-on sema3A. In this case CD25^high^FoxP3^high^ Breg cells were characterized by being IL-10^high^ whereas FoxP3^dim^ B cells were IL-10^dim^ as well. When analyzed in SLE patients, we found CD19^+^CD25^high^FoxP3^high^ cells to be significantly increased as compared to healthy individuals. This was found to be in positive correlation with increased SLEDAI and in association with lupus nephritis. In a recent study and in line with our finding, CD19^+^CD25^high^FoxP3^high^ B regulatory cells were found to be increased in the cerebrospinal fluid of active patients suffering from relapsing-remitting multiple sclerosis (MS) when compared to that of nonclinically active MS. This expansion of B regulatory cells was attributed to the compensatory attempt of these cells to maintain immune regulatory processes [21]. In contrast to this study, rheumatoid arthritis patients had significantly lower proportions of peripheral blood CD19^+^FoxP3^+^ B cells as compared to healthy controls, particularly in patients with interstitial lung disease. This finding suggests that Breg phenotypes may have different functions in the pathogenesis of different rheumatic diseases [22]. The fact that serum IL-10 is increased in SLE and in association with SLE disease activity has been established in many previous studies. In one, increased IL-10 was shown to exhibit a modulatory effect by suppressing the differentiation and function of monocyte-derived dendritic cells [23]. In a recent study, increased IL-10 in the sera of SLE patients was capable of inducing Fas and FasL expression on CD4^+^ T cell surfaces, promoting apoptosis of this cell subset, thus contributing to many other mechanisms of self-tolerance [24]. However, we still need to explain the mechanisms by which serum IL-10 is increased in SLE. In this regard, the expansion of IL-10 producing B cells was shown to be in part the result of increased B cell activating factor (BAFF). Enhanced serum BAFF in SLE was described in many studies as being associated with increased expression of TLR-9 and other markers of B cell activation [25, 26]. This may explain our finding of increased IL-10^high^FoxP3^high^ Breg cells as well as increased serum IL-10 in SLE. Another significance of FoxP3^high^ B cells being increased in SLE is the possibility that by multiplying they also increase their IL-10 production improving by this their regulatory function. When B cells were cocultured with sema3A they responded by increasing their FoxP3 expression. This raises the possibility that if provided with the proper stimulation Bregs may develop higher regulatory properties and that by increasing their IL-10 production they may induce a better regulatory mechanism in SLE.

## 5. Conclusion

CD25^high^FoxP3^high^ Bregs (highly expressing IL-10) are significantly increased in SLE, in correlation with SLEDAI. Semaphorin 3A increases FoxP3 expression in Breg cells improving by this their regulatory properties. We assume that the expansion of these cells is the attempt of our regulatory immune responses to maintain self-tolerance and to suppress as much as possible SLE disease activity. Further studies are required in order to better understand the role of this subset of B cells in autoimmunity.
